# Adaptive responses of androgen receptor signaling in castration-resistant prostate cancer

**DOI:** 10.18632/oncotarget.4689

**Published:** 2015-06-29

**Authors:** Sven Perner, Marcus V. Cronauer, Andres Jan Schrader, Helmut Klocker, Zoran Culig, Aria Baniahmad

**Affiliations:** ^1^ Section for Prostate Cancer Research, Institute of Pathology, Center for Integrated Oncology Cologne/Bonn, University Hospital of Bonn, Bonn, Germany; ^2^ Department of Urology, Ulm University Medical Center, Germany; ^3^ Department of Urology, Muenster University Medical Center, Germany; ^4^ Division of Experimental Urology, Department of Urology, Medical University of Innsbruck, Austria; ^5^ Department of Urology, Medical University of Innsbruck, Austria; ^6^ Institute of Human Genetics, Jena University Hospital, Germany

**Keywords:** androgen receptor, prostate cancer

## Abstract

Prostate Cancer (PCa) is an important age-related disease being the most common cancer malignancy and the second leading cause of cancer mortality in men in Western countries. Initially, PCa progression is androgen receptor (AR)- and androgen-dependent. Eventually advanced PCa reaches the stage of Castration-Resistant Prostate Cancer (CRPC), but remains dependent on AR, which indicates the importance of AR activity also for CRPC. Here, we discuss various pathways that influence the AR activity in CRPC, which indicates an adaptation of the AR signaling in PCa to overcome the treatment of PCa. The adaptation pathways include interferences of the normal regulation of the AR protein level, the expression of AR variants, the crosstalk of the AR with cytokine tyrosine kinases, the Src-Akt-, the MAPK-signaling pathways and AR corepressors. Furthermore, we summarize the current treatment options with regard to the underlying molecular basis of the common adaptation processes of AR signaling that may arise after the treatment with AR antagonists, androgen deprivation therapy (ADT) as well as for CRPC, and point towards novel therapeutic strategies. The understanding of individualized adaptation processes in PCa will lead to individualized treatment options in the future.

## INTRODUCTION

Prostate cancer (PCa) is the most common cancer type among men in Western countries and the second leading cause of cancer-related death in males [1]. In more than 80% of cases, PCa is diagnosed at the local stage and is often a low-risk disease with indolent clinical course and favorable survival [2]. The management of localized PCa is controversial as the identification of the subset of patients with aggressive, high-risk disease remains challenging. Considering histopathological features (i.e. Gleason grade, extent of the tumor), serum prostate specific antigen (PSA) level, clinical stage, age and co-morbidity of the patient, treatment of localized PCa includes active surveillance, radical prostatectomy or radiotherapy [2, 3].

Local recurrent PCa after failure of primary surgery/radiotherapy and hormone-naive metastatic disease are treated with androgen deprivation therapy (ADT) [4]. Luteinizing hormone-releasing hormone (LHRH) agonists or LHRH antagonists, eventually combined with anti-androgens as bicalutamide, are used to achieve hormonal depletion [4]. Initially, the reduction of circulating androgens decreases androgen receptor (AR)-mediated proliferation and survival of tumor cells. Therefore, ADT leads to remission of the tumor lasting for up to a few years and results in a decline of serum PSA [5]. However, after initial response to ADT, tumor cells ultimately become castration-resistant resulting in progression of the disease despite anorchid serum androgen levels [6]. Of all patients diagnosed of PCa, 10-20% develop castration-resistant disease, mostly within a few months to a few years after initiation of ADT [7].

The underlying molecular basis how PCa cells escape from the growth control by exogenous androgens is still poorly understood. Adaptive mechanisms of PCa cells include molecular alterations in response to androgen ablation leading to re-activation of the AR despite low circulating androgens after initial response to ADT [8]. Over the past years, several studies give evidence for selection advantages resulting in clonal outgrowth of initial androgen-independent cells [9]. This theory of castration-resistance is based on the observation that aggressive castration-resistant cells show distinct gene expression patterns and molecular properties, which are not present in parental PCa cells [10].

Mechanisms mediating castration-resistance comprise the re-activation of AR signaling despite low levels of circulating androgens on the one hand, as well as the activation of alternative AR-independent pathways on the other hand.

Maintained AR activity under androgen deprivation is based on genetic and functional aberrations affecting components of the AR signaling axis which have been observed in CRPC cells [11]. Genetic alterations such as AR gene amplification resulting in increased AR expression occur in about one third of CRPC tumors [11, 12], while AR mutation or alternative splice variants allowing the tumor to respond to very low androgen levels can be observed in a smaller subset of CRPC cases [11, 13]. Persistent transcriptional AR activity can also be mediated through alternative ligands as progesterone or by ligand-independent transcriptional activity [14]. Furthermore, the intratumoral conversion of androgen precursors as well as the *de novo* steroidogenesis provides persistent intraprostatic androgen concentrations sufficient to activate the AR despite low serum testosterone [15]. Additionally, alternative AR activation eventuates from alterations of coactivators and corepressors of the AR signaling as well as cross-activation through bypass pathways [16, 17]. Genetic alterations frequently found in CRPC contributing to dysregulated survival signaling involve c-myc amplification, PTEN loss, as well as alterations of genes implicated in the growth factor receptor signaling such as PI3K, Src kinase, Ras/MAPK [6, 8].

Admittedly, no treatment options with curative intent are available for castration-resistant prostate cancer (CRPC) to date [4]. Current palliative therapeutic strategies for CRPC comprise docetaxel as conventional chemotherapy, the immunostimulant sipuleucel-T and the inhibitor of androgen synthesis abiraterone acetate [4]. Cabazitaxel as chemotherapeutic agent, the AR antagonist enzalutamide and the radiopharmaceutical radium-223 are available as second line therapy after docetaxel treatment [4]. Additional options for patients with metastatic CRPC include the bone-targeting agents zoledronic acid and the receptor activator of nuclear factor kB ligand inhibitor denosumab [4]. Various pre-clinical approaches identified promising strategies to prevent rapid progression to castration-resistance [18]. Different classes of agents targeting components involved in survival pathways [19], DNA damage repair [20], angiogenesis [21], tumor microenvironment [22] or the immune system [4, 18] have reached phase III in clinical trials. Future studies will reveal whether these agents have the potential to significantly increase survival of patients with CRPC.

This review will highlight the current knowledge about adaptive mechanisms of the AR signaling as well as the significance of its interaction partners contributing to the development of castration-resistance.

## ADAPTIVE RESPONSES IN AR SIGNALING THROUGH AR MUTANTS AND AR VARIANTS

**Figure 1:**
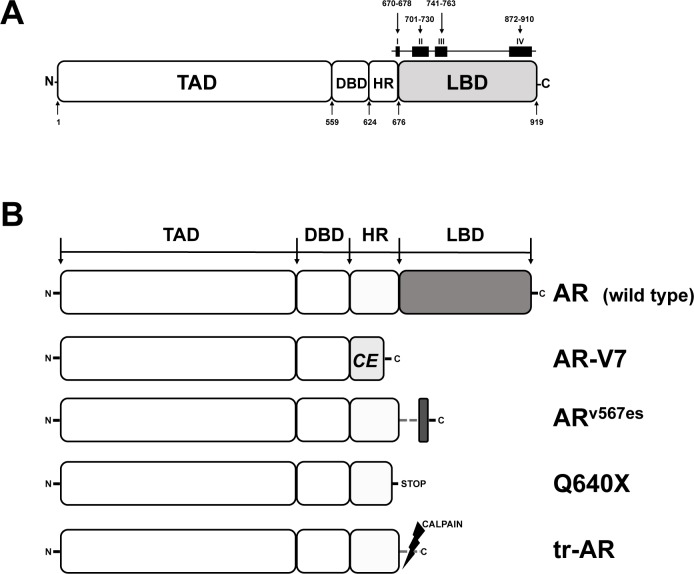
Functional domains of the human AR and AR variants expressed in PCa **A.** Functional regions of the AR. TAD, transactivation domain; DBD, DNA-binding domain; HR, hinge region; LBD, ligand binding domain. Squares (I-IV) on top of the LBD delineate clusters of AR mutations, numbers indicate amino acid (aa) positions. **B.** AR and AR∆LBD variants identified in PCa. AR, full length AR wild type; AR-V7, product of alternative splicing, CE, new cryptic exon; ARv567es, product of altered splicing, exon 5, 6, 7 skipped during splicing; Q640X, AR with a nonsense mutation leading to a truncated AR of 640 aa; tr-AR, truncated AR, enzymatically cleaved by calpain.

An adaptive response in AR signaling may also occur at the level of mutations and splice variants of the AR that occur under the selective pressure of ADT. Structurally, the AR is organized in 4 different domains: the N-terminal transactivation domain (TAD), a central DNA-binding domain (DBD), a hinge region (HR) which connects the DBD to the carboxy-terminal ligand binding domain (LBD) (Figure 1A). Upon androgen binding, the AR dissociates from heat shock proteins (HSP) and translocates to the nucleus where it dimerizes with another AR molecule. Subsequently, this AR dimer binds to chromatin and androgen response elements in the promoter regions of androgen-dependent genes, thereby activating/inhibiting their transcription. The implication of HSP in adaptation of AR signaling in PCa has recently been reviewed and suggests an increased expression of HSP70 and HSP27 that correlates with PCa aggressiveness and CRPC [23].

AR mutations are very rare in early stages of PCa. However, approximately 10-30% of CRPC patients carry AR mutations, especially when treated with ADT, indicating an adaptation to ADT by changing AR function [24]. In CRPC almost 50% of AR mutations cluster to 4 discrete regions of the AR LBD (Figure 1A). Somatic mutations in the AR LBD usually result in decreased receptor specificity, thereby broadening the number of steroids that can bind and activate the receptor. In addition, many of these mutated AR can be activated by anti-androgens. A prototype for this promiscuous gain of function mutants is AR-T877A. Initially identified in LNCaP cells, T877A (now T878A, according to the AR Mutations Database at http://androgendb.mcgill.ca, [25] was repeatedly found in flutamide-treated CRPC patients [26-28]. Functional studies demonstrated that T877A is strongly activated by the anti-androgens flutamide/hydroxyflutamide and by progesterone. Interestingly, the CYP17A1 inhibitor abiraterone was shown to increase intracellular progesterone levels, thereby allowing progesterone-inducible T877A to circumvent abiraterone-mediated inhibition of AR signaling in CRPC cells [29]. While T877A diminishes the efficacy of abiraterone, another interesting mutation, F876L, is able to convert AR antagonists like enzalutamide and ARN-509 to AR agonists [30, 31]. Most importantly, F876L still remained sensitive to the effects of bicalutamide [30].

Early functional *in vitro* studies showed a high constitutive transcriptional activity of AR constructs in which the LBD has been artificially deleted [32]. Due to the deletion of the functional LBD situated in the AR C-terminus, these AR variants are generally referred to as AR∆LBD. Blocking of the androgen/AR signaling axis was shown to induce a rapid increase of AR∆LBD in PCa cells [33]. So far, 17 AR∆LBD variants have been isolated from castration-resistant tumor cell lines/xenografts or clinical tumor specimens. Although AR∆LBD are predominantly products of alternative splicing (AR-V), they can also be products of nonsense mutations (AR-Q640X) or proteolytic cleavage (tr-AR) [34] (Figure 1B). Although all AR∆LBD lack a functional LBD, they can be subdivided into 2 structurally different subgroups, depending on the presence or absence of a HR (Figure 1B). Besides its function as a flexible linker between the DBD and the LBD, the HR carries a nuclear localization signal and a microtubule binding domain.

As AR∆LBD do not express a functional LBD, they are insensitive to all currently available hormonal therapies targeting directly (anti-androgens) or indirectly (inhibitors of androgen synthesis) the LBD [35, 36]. These *in vitro* observations could explain at least in part the well described cross-resistance between abiraterone and enzalutamide in the clinical setting [37, 38]. In addition, there is experimental evidence that different AR∆LBD variants are able to determine the sensitivity in PCa cells towards first generation taxanes like docetaxel or paclitaxel [39]. Both compounds were shown to impair nuclear localization of the AR via modulation of the microtubule AR network [39, 40]. As suggested by Thadani-Mulero, only AR forms expressing a microtubule binding domain situated in the HR of the receptor (e.g. wild type AR, AR^v567es^) are susceptible to taxane-mediated microtubule stabilization that is abrogating nuclear translocation and transcriptional activity of these receptors [40]. In contrast, HR-negative AR∆LBD like AR-V7 that do not associate with the microtubule machinery accumulate in the nucleus via a yet unknown mechanism thereby activating the transcriptional machinery [40]. However, the ability of taxanes to modulate AR/AR∆LBD-signaling in the clinical setting has been discussed with some controversy [41, 42]. Enzalutamide-resistant tumors often exhibit a cross-resistance with docetaxel. By contrast, cabazitaxel, a second generation taxane, remains highly effective in enzalutamide-resistant tumors, indicating that the inhibitory effects of docetaxel on AR/AR∆LBD-signaling represent only a minor part of its antitumor activity [41]. Moreover, the concentrations reported to affect AR and AR∆LBD translocation *in vitro* [40] are far beyond the effective taxane concentrations achieved in the clinical setting [41, 42]. As a result, a more thorough analysis of the mechanisms involved in regulation of AR/AR∆LBD-signaling by taxanes is needed.

The occurrence and detection of AR point mutations and AR∆LBD are of prognostic and therapeutic significance. In order to guide initial treatment selection or sequential therapies, there is an urgent need for new markers. Serum biomarker studies may be difficult to compare because of different procedures in various laboratories. Current research is focusing on the non-invasive retrieval of tumor DNA from blood/serum samples, i.e. circulating tumor DNA (ctDNA) or DNA from circulating tumor cells (CTC) to analyze aberrant AR variants. AR mutants like F876L or T877A have already been successfully isolated from blood and CTC samples isolated from patients suffering from advanced CRPC [31, 43, 44]. Recently, Antonarakis et al. were able to demonstrate that patients with AR-V7 expression in CTCs had statistically shorter time to PSA and radiographic progression and shorter overall survival [45]. These findings are supported by a recent study from our group, which analyzed both AR-V7 and AR point mutations in CTCs of patients suffering from advanced PCa [44].

The discovery of constitutively active AR∆LBD that do not express a LBD has led to the development of promising novel experimental approaches targeting AR-signaling in a LBD-independent manner [46-51]. Recent efforts to develop drugs targeting the TAD situated in the N-terminus of the AR have led to the discovery of the small molecule inhibitor EPI-001, a bisphenol A-derivative that binds covalently and inhibits the AR amino-terminal TAD [46]. Low toxicity combined with the ability to block transactivation of both AR and AR∆LBDs makes EPI-001 the most promising third generation compound for the treatment of CRPC.

The analysis of AR-point mutations and/or AR-splice variants has led to the discovery of new prognostic and therapeutic targets in CRPC. In summary, the combination of new prognostic parameters able to guide treatment selection along with novel therapeutic approaches will establish a new era of personalized, targeted therapies.

## ADAPTIVE RESPONSES IN AR SIGNALING BY TRANSLATIONAL REGULATION OF THE AR

The upregulation of AR protein is a hallmark of CRPC and seems to represent an adaptive response to ADT [52]. Presumably, the increased AR protein level expedites the reactivation of the AR signaling. On the one hand, the AR gene amplification is observed in about one third of these cases [53] as one mechanism while on the other hand post-translational regulation represents another underlying molecular mechanism with increasing importance for enhancing AR signaling. Here the factors and pathways at the level of translational regulation of the AR are described in the context of PCa. Interestingly, the AR transcript has a long 3′untranslated region (3′UTR), which is predestinated for post-translational regulation via RNA binding proteins that modulate mRNA stability or translation efficiency. RNA binding proteins Hu antigen R and polyC-binding proteins (PCBPs) 1 and 2 were found to bind to a UC rich motif in the 3′UTR of AR mRNA and regulate its translation [54, 55]. PCBP1 was identified as a blocker of AR translation in dedifferentiating endometrial cells and this role was confirmed in LNCaP PCa cells [56].

An UC-rich 3′UTR motif is also the target site of another AR post-transcriptional inhibitor, ErbB3 binding protein (EBP1) that in addition also binds to a RNA stem-loop formed by the CAG repeats encoding the poly-glutamine stretch in the AR N-terminus [57]. Whereas EBP1 interaction with the UC region in the 3′UTR promotes mRNA decay, its binding to the CAG stem-loop seems to attenuate translation of AR mRNA. This post-transcriptional inhibition of AR protein synthesis of EBP1 is an additional function to its described activity as a repressor protein of AR transcriptional activity [58, 59]. In line with its interference with the AR axis, EBP1 was found downregulated in advanced stages of PCa [60] and inhibiting PCa cell growth when overexpressed [58].

The nucleic acid binding protein called heterogeneous nuclear ribonucleoprotein K (hnRNP-K) is an additional inhibitor of AR mRNA translation and an inhibitor of PCa cell proliferation via binding to several sites in the AR mRNA including sites in both untranslated terminal regions and in the coding region [61]. Further support for its inhibitory role in PCa comes from its inverse correlation with AR protein in primary prostate tumors and its altered pattern of expression in tumor metastases.

A mechanism for enhancement of AR translation was identified recently and is based on a reciprocal link between the AR and the ribonucleoprotein transcriptional enhancer complex assembled by midline 1 (MID1), a protein that is mutated in the developmental syndrome Opitz G/BBB [62, 63]. Overexpression of MID1 in PCa cells results in an upregulation of AR protein and in line with this an increase of AR transcriptional activity whereas MID1 knockdown decreases AR protein levels [64]. Within the AR N-terminus there is a poly-glutamine and a poly-glycine repeat both encoded by purine-rich trinucleotid repeats, a CAG and a GGY repeat, respectively. Notably, both trinucleotide repeats interact with the MID1 protein complex. The MID1 protein complex binds the AR mRNA via both trinucleotide repeats and enhances AR translation [64]. In addition to MID1, this transcriptional regulator complex contains the regulatory and the catalytic subunits of protein phosphatase 2A (alpha-4 and PP2Ac) [65] and MID1 also has an ubiquitin ligase activity that targets PP2Ac in the complex to proteasomal degradation thus regulating PP2A activity [66]. As PP2A is a major antagonist of protein kinases involved in growth factor signaling cascades, its degradation further stimulates tumor cells [67].

AR regulation by MID1 occurs via enhancement of AR translation. However, the interaction of the MID1 complex and the AR axis is not uni- but bi-directional. Whereas MID1 regulates AR protein through translation control, AR is a negative regulator of MID1 via several AR binding sites in the MID1 gene. This mutual interaction forms a classical regulatory feedback loop that was suggested as a fine-tuning mechanism for homeostasis of AR protein level. ADT of PCa would disrupt this mechanism and result in MID1 and subsequently AR protein upregulation. In line with this, MID1 is significantly overexpressed in PCa in a stage-dependent manner [64].

Noteworthy, the assembly of the MID1-alpha4/PP2Ac protein complex is disrupted by the antidiabetic drug metformin as first reported by Kickstein et al. [68]. Besides decreasing blood glucose levels metformin inhibits many types of cancer and non-malignant cells, among them also PCa cells [69, 70]. In addition it was suggested as a tumor preventive drug, although these data are still inconclusive [71-74]. Testing its effect on the MID1-AR feedback loop revealed disruption of AR mRNA association with the protein complex and subsequent downregulation of AR protein in PCa cells treated with metformin [75]. The inhibitory effect of metformin was mimicked by disruption of the MID1-alpha4/PP2As protein complex by siRNA knockdown of MID1 or alpha4 whereas activation of another target of metformin, AMP kinase was not required.

Thus, the inhibition of AR protein levels by metformin suggests its use in the treatment of hormone-naïve PCa and CRPC. In support of this, a metformin treatment study in 44 men with progressive metastatic CRPC who received metformin until disease progression resulted in disease stabilization and prolongation of PSA doubling time [76]. Retrospective analysis revealed that the risk of progression to CRPC in patients treated for localized disease with external-beam radiation therapy was reduced in metformin users compared to patients treated with other anti-diabetic drugs [77]. In contrast another recent study did not confirm a reduced risk for adverse outcome in PCa patients by metformin alone [78], which calls for further thorough evaluation of a potential benefit of metformin in the treatment of PCa. Perhaps, the combination of metformin and AR antagonists, such as enzalutamide, that is currently under clinical investigation (NCT02339168), may provide a benefit for patients.

Last but not least, a new class of post-transcriptional regulators also acting on AR, are micro-RNAs (miRNAs) that control gene expression by inhibition of protein translation or induction of mRNA cleavage. These small RNAs of about 22 bases in length are generated by processing of mostly untranslated RNA and each miRNA can regulate a variety of target mRNAs [79, 80]. Several miRNAs have been reported to target various sites in AR mRNA and inhibit androgen receptor-positive PCa cell lines, e.g. miR488*, miR125, miR205, mir185, miR1, miR31 [81-86]. A systematic combined experimental and *in silico* screen for miRNAs targeting the long 3′UTR of the AR performed in PCa cell lines identified 75 miRNAs that regulate AR protein level [87]. Fifteen miRNAs downregulating AR were confirmed to decrease androgen-induced proliferation of PCa cells. This number underscores the complexity of post-transcriptional regulation by miRNAs and offer new strategies for therapeutic intervention.

In conclusion, the diverse mechanisms of AR modulation at the level of mRNA and translation into protein can result in adaptation of AR signaling. The variety of proteins and miRNAs involved define a complex regulatory network for fine-tuning of AR protein level in PCa that might be used as drug targets and calls for efforts to develop methods to interfere with post-transcriptional AR regulation for improving inhibition of the AR axis in PCa therapy.

## ADAPTIVE RESPONSES IN AR SIGNALING THROUGH GROWTH FACTORS AND TYROSINE KINASES

There are several levels of interaction between growth factor receptors and AR in PCa. Transcription activation function of the AR could be enhanced by growth factors and growth factor-related receptors in a synergistic and ligand-independent manner [88, 89].

Mechanistically, growth factors and related receptors lead to activation of MAPK that phosphorylate specific amino acids in the N-terminal region of the AR [90]. In consequence, HER-2, which activates kinases of the MAPK group, leads to AR-dependent progression of PCa, as evidenced in the LAPC-4 model [91]. The effect of HER-2 on AR could be explained by modulation of receptor DNA binding and AR stability [92]. Activation of the AR by HER-2 and HER-3 was reported also in a PCa recurrent cell line CWR-R1 [93]. Induction of HER-2 occurs *in vitro* in conditions of androgen depletion and pointing to possible adaptive and compensatory effects of endocrine therapy [94]. Consistently with these observations, HER-2 expression is increased during cancerogenesis of PCa and leads to elevation of expression of the AR downstream gene PSA [95, 96]. In addition, compensatory upregulation of the Etx/BMX tyrosine kinase was observed in CRPC cells [97].

MAPK are implicated in activation of the AR by interleukin-6, a cytokine whose expression is elevated in human PCa [98]. It was also demonstrated that interleukin-6 activation of the AR occurs through activation of MAPK and phosphorylation of N-terminal amino acids [99]. Notably, the tyrosine kinase Src and FAK are implicated in regulation of growth and migration of PCa cells by interleukin-6 [100]. Interleukin-6 is a positive growth factor being responsible for inhibition of apoptosis and angiogenesis in several human cancers, including PCa. Similarly, tyrosine kinase Pim1 and Etk are required for AR activation by interleukin-6 [101]. AR coactivators p300 and SRC-1 are particularly important for AR activation by IL-6 [102, 103]. Both coactivators are highly expressed in prostate cancer and are identified as targets for therapy [104-106]. After IL-6 binding to the receptor, signal transducer and activator of transcription (STAT)3 factor is translocated to the nucleus and phosphorylated. Although in LNCaP cells phosphorylation of STAT3 may be associated with either growth inhibition or stimulation, there is evidence obtained with other models according to which STAT3 is a valid target for therapy [107]. AR activation by IL-6 is potentiated by STAT3 and MAPK pathways [108] as evidenced by association of STAT3 with the AR, which occurs in an IL-6-dependent manner [109].

Taken together, the results of several studies mentioned above have indicated that tyrosine kinases are particularly important in AR activation in conditions in which the levels of circulating androgen are diminished during therapy, indicating an adaptation response.

There is also a link between AR mutations and growth factor receptors. Androgens and hormones that activate mutated AR in LNCaP cells increase expression of epidermal growth factor receptor expression [110]. These findings could be explained by association between AR and epidermal growth factor receptor [111]. Proliferative effect of androgens and epidermal growth factor could also be explained by their down-regulation of the cell cycle inhibitor p27 [112].

Because of these findings one can propose the development of preclinical and clinical inhibitors of tyrosine kinases to inhibit the crosstalk between growth factor receptor and AR signaling pathways. Treatment of cells with an anti-androgen and the anti-HER2 receptor monoclonal antibody herceptin could open the way for novel PCa therapies. In line with these observations, the dual ErbB1/ErbB2 tyrosine kinase inhibitor PKI-166 was tested in series of human PCa xenografts [113]. The effects of the inhibitor could be abolished by androgenic administration [113].

In context of crosstalk between signaling pathways, further aspects of inhibition of tyrosine kinases by sorafenib, a multikinase inhibitor, have to be discussed. Sorafenib inhibits proliferation and induced apoptosis in several PCa cell lines [114]. Sorafenib is a multikinase inhibitor that is approved for therapy of renal and liver cancer. Androgen-sensitive PCa cells were inhibited to a higher extent compared to androgen-insensitive ones. It was demonstrated that several targets of sorafenib, which were identified previously, are also inhibited in PCa cells. It was also demonstrated that cell lines that become resistant to endocrine and chemotherapy in PCa are at least partially responsive to sorafenib. A higher sensitivity of AR-positive cell lines to sorafenib could be explained by down-regulation of AR expression.

These *in vitro* findings could also have clinical implications. Although PCa is a heterogenous disease, patients with higher expression of AR and several other sorafenib targets may be good candidates for sorafenib treatment. Thus, preclinical results obtained with sorafenib may serve as a basis for the development of a more personalized approach for PCa patients.

In summary, there are multiple interactions between signaling pathways that include tyrosine kinase receptors and the AR. These interactions are basis for a rational therapeutic targeting of these interactions in clinics.

## ADAPTIVE RESPONSES IN AR SIGNALING THROUGH THE ACTIVATION OF THE SRC-AKT - AND MAPK-PATHWAYS INACTIVATE AR-COREPRESSORS AND LEAD TO ENHANCED CANCER PROGRESSION

The AR signaling is not only controlled by the ligands of AR but also by signal transduction pathways including the MAPK and non-receptor tyrosine kinases. Among tyrosine kinases, Src is particularly important for AR phosphorylation [115]. In PCa, a correlation between AR tyrosine phosphorylation and Src tyrosine kinase activity was observed. Importantly, AR activation by Src was confirmed in CRPC cells [116, 117].

Intracellular kinases that mediate the signal transduction of membrane associated receptor tyrosine kinases are often linked to cellular growth and seem to play a critical role in cancer development and progression. The family of non-receptor tyrosine kinases, including Src, are activated by and interact with various cellular pathways and regulate a plethora of different pathways including cell proliferation, cell motility, invasion, epithelial-to-mesenchymal transition, resistance to apoptosis, and metastatic spread (reviewed in: [118, 119]. Src expression is upregulated in human CRPC cells [116, 118, 120]. Several lines of evidence indicate that the expression of Src and Src kinase family members can drive the formation of PCa or the progression to CRPC [118]. Interestingly the interaction of AR with Src has been shown to lead to AR activation by phosphorylation of AR [115].

Another underlying molecular basis to activate the AR by the Src family has been suggested to be mediated through inactivation of corepressors. Corepressors are transcriptional regulators that interact with DNA-bound transcription factors and lead to inhibition of their transactivation. Some corepressors interact with the amino-terminus of AR and some with the DBD, which may be used to reduce the transcriptional activity of AR point mutations and AR∆DBD isoforms [121].

In the presence of AR antagonists (anti-androgens), the corepressor recruitment leads to recruitment of chromatin repressor complexes to AR target genes or to coactivator displacement on the AR. These mechanisms are the molecular basis for AR inactivation mediated by some AR antagonists [121-123]. In addition to antagonist induced corepressor binding to AR, LCoR, a ligand-dependent corepressor for AR, reduces AR activity in the presence of androgens including dihydrotestosterone [117]. In contrast to non-PCa cells, LCoR has only weak repression function in CRPC cells indicating an adaptive response in CRPC to inactivate corepressor function. Therefore, combination therapy of inhibitors against Src and Src family members and against the AR may be fruitful.

To analyze the underlying molecular mechanism, a battery of various inhibitors of signaling pathways were used to treat CRPC cells. The results indicated that the LCoR silencing function is repressed by the Src-Akt pathway [117]. Inhibition of Src signaling represses AR target gene expression indicating that the Src family members enhance the AR transactivation at chromatin level. In the presence of a Src inhibitor, LCoR is more potently recruited to AR target genes and reduced the expression such as that of PSA. The Src-LCoR-AR pathway was confirmed by LCoR expression and inactivation of Src to regulate CRPC tumor growth of human xenografts in mice. Thus, the Src-Akt signaling inactivates LCoR, which subsequently activates AR signaling in human CRPC cells in culture and in mouse xenograft model [117].

These findings and those from other groups strongly suggest that inhibitors of members of the Src tyrosine kinase family in combination therapy with antagonists may be a very useful tool to inactivate AR signaling and progression of CRPC, which has been nicely reviewed recently [124]. In line with this combinatorial treatment idea, the use of PI3K inhibitors is currently under clinical investigation in combination with AR antagonists such as enzalutamide (UKCRN Study ID: 16580).

Similarly to LCoR, the corepressor Silencing Mediator for Retinoid and Thyroid hormone receptors (SMRT) seems also to be inactivated by signaling pathways in PCa. An activated MAPK signaling was shown to inhibit the SMRT corepressor function [125]. SMRT binds to the AR in the presence of AR antagonists and inactivates the AR-mediated transactivation. SMRT is recruited to AR binding sites at chromatin and recruits the SAP30-SIN3A-HDAC repressor complex [126]. In addition SMRT competes with the coactivator SRC1 for the binding to AR [127]. However in PCa cells, SMRT exhibited only little silencing activity. Analyses of various signaling pathways suggest that the MAPK is one major pathway that inactivates SMRT repressor function. Using an ERK1/2 specific inhibitor, the binding of SMRT to AR was enhanced as well as the chromatin recruitment of SMRT to the PSA gene was increased. Furthermore, a synergy by the co-treatment of the AR antagonist and ERK1/2 inhibitor revealed a potent inhibition of PCa cell growth and colony formation [125]. These findings strongly suggest that the MAPK inactivates AR corepressors and thereby activates the AR signaling as an adaptive response. These observations also suggest that inhibitors of the MAPK pathway [128] in combination therapy with AR antagonists may be a very useful tool to inactivate AR signaling and progression of PCa.

Thus, mitogenic signaling pathways activate the AR signaling. One underlying molecular basis is the inactivation of AR corepressors by the Src kinase family members and/or the MAPK signaling. Co-treatment with signal transduction inhibitors may be a useful tool and therapeutic approach to inhibit CRPC. Presumably tissue specific inhibitors of Src, PI3K, Akt and MAPK are more useful in combination with AR inhibitors to reduce side-effects.

**Figure 2:**
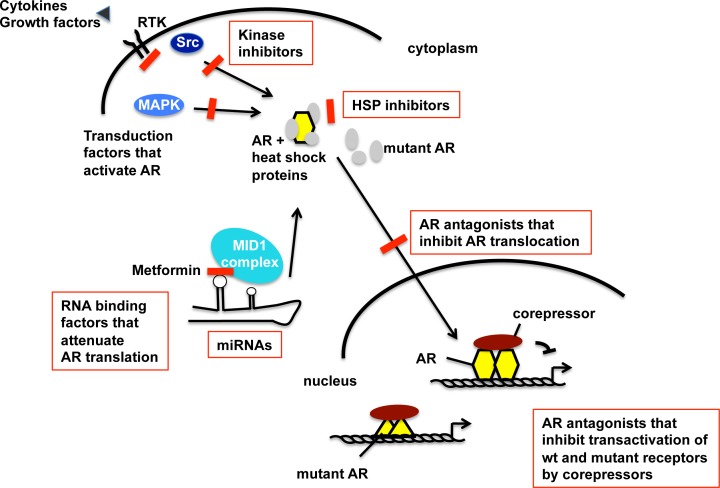
Schematic view of the AR activation pathway that undergoes adaptation during progression towards CRPC and therapy resistance Summarized targets for future therapeutic interventions of CRPC within the AR signaling pathway and AR adaptive responses. The level of increased AR protein in CRPC is in part regulated at mRNA level. In addition to its cognate hormone, the AR is activated by tyrosine kinase and MAPK signaling, leading to dissociation of heat shock proteins (HSP). HSPs might thus present targets of intervention into AR signaling [23]. Further steps of AR signaling are the translocation to the nucleus, DNA binding and regulation of AR target gene expression. Besides the wild-type (wt) AR, isoforms and mutants of AR exist for which the inhibition is also an important future goal for treatment of CRPC. Detection of activated individualized pathways that activate the AR may allow using highly specific inhibitors in combination therapies. Inhibitors of specific AR activation signaling are highlighted in red color.

Taken together, multiple adaptation processes seem to exist that allow persistent AR signaling in PCa (Figure 2). The detailed knowledge about possible pathways that lead to the activation of the AR allows to detect individualized adaptation processes in PCa and consequently allows an optimal individualized treatment option.

## Abbreviations

ADT: androgen deprivation therapy
AR: androgen receptor
CRPC: castration resistant prostate cancer
CTC: circulating tumor cells
DBD: DNA binding domain
HR: hinge region
LBD: ligand binding domain
LHRH: luteinizing hormone-releasing hormone
mCRPC: metastatic CRPC
MAPK: Mitogen-activated protein kinases
PCa: prostate cancer
PSA: prostate specific antigen
SMRT: silencing mediator of retinoic acid and thyroid hormone receptor
TAD: transactivation domain
wt: wild-type
